# Getting Ahead of the Curve: How Ochsner Became a Leader in SARS-CoV-2 Diagnostic Testing

**DOI:** 10.31486/toj.20.0055

**Published:** 2020

**Authors:** Luke J. Caruso, Donald D. Chang, Karrie B. Hovis, Andrea J. Linscott, Earlene F. Goens, Tong Yang, Elise Occhipinti

**Affiliations:** ^1^ Department of Pathology and Laboratory Medicine, Ochsner Clinic Foundation, New Orleans, LA; ^2^ The University of Queensland Faculty of Medicine, Ochsner Clinical School, New Orleans, LA

On March 9, 2020, Ochsner Health admitted its first COVID-19 patient. Less than 2 weeks later, Ochsner became the first hospital in Louisiana to insource diagnostic testing for severe acute respiratory syndrome coronavirus 2 (SARS-CoV-2), the virus responsible for COVID-19. While Ochsner soon became widely recognized for leading the state in SARS-CoV-2 testing, few were aware of the preparations that occurred beforehand. Here, we share the incredible journey of how Ochsner's laboratory team was able to rapidly respond and deliver diagnostic testing that proved essential to improving patient care in the state of Louisiana (Figure).


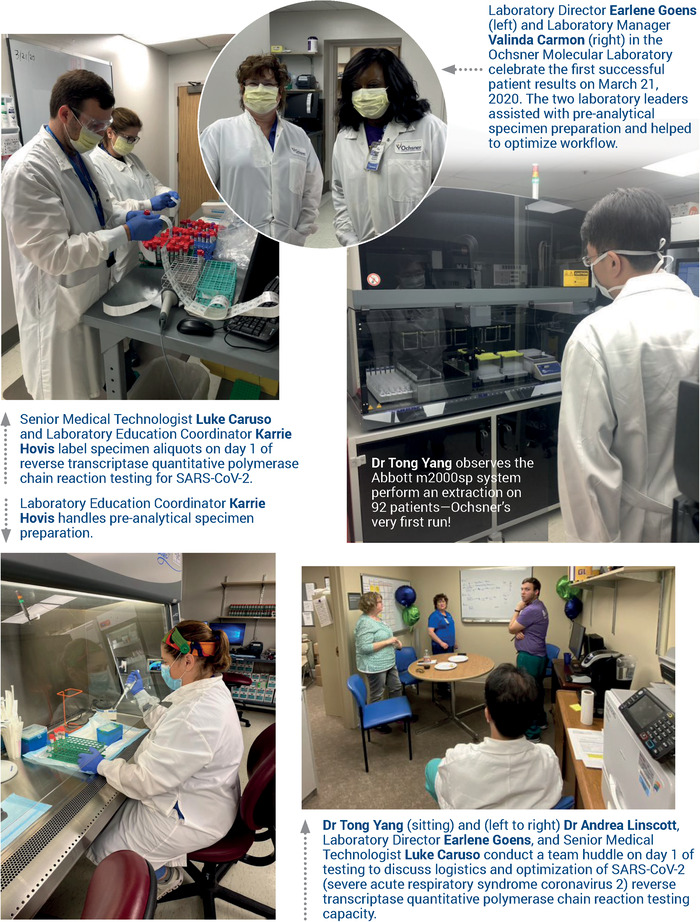



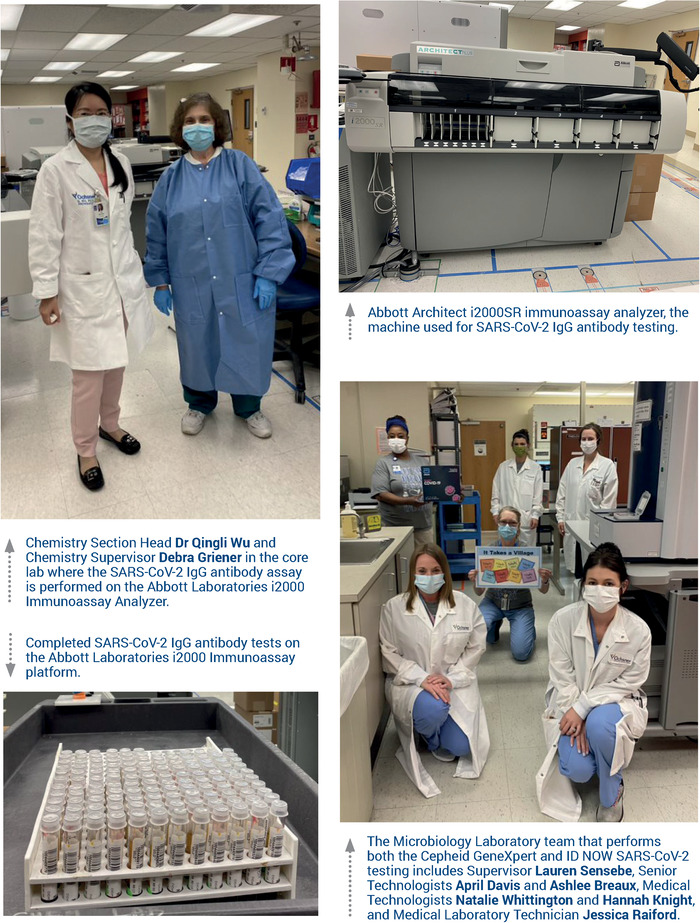


**Figure.**
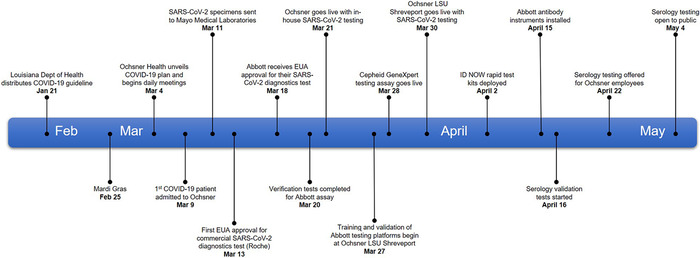
**Timeline of diagnostic testing implementation at Ochsner Health in New Orleans and Shreveport, LA (February to May 2020).** EUA, emergency use authorization. SARS-CoV-2, severe acute respiratory syndrome coronavirus 2.

Months before the pandemic affected New Orleans, various departmental leaders began weekly meetings to monitor the emerging threat of a novel coronavirus, later identified as SARS-CoV-2. Beginning in late January, a committee consisting of leaders from systemwide management, infection control, infectious diseases, information systems, laboratory, nursing, security, and supply chain met weekly to review data and recommendations from the Centers for Disease Control and Prevention (CDC). The teams began to formulate strategies for the protection of frontline staff, infection control, patient management, and, of utmost importance to the lab, criteria for testing for the SARS-CoV-2 virus.

In late February, the US Food and Drug Administration (FDA) issued guidance for an emergency use authorization (EUA) for SARS-CoV-2 molecular testing. An EUA is an expedited review process applied during an emergency that allows medical products to be used clinically even though they have not gone through the formal FDA approval process. At the time, the main SARS-CoV-2 diagnostic test used a molecular method called reverse transcriptase quantitative polymerase chain reaction (RT-qPCR), an approach that detects the viral RNA and, if detected, amplifies it into a signal.

When the first cases emerged in Louisiana, a few independent vendors and federal testing centers had received EUA approval, but no commercial labs had EUA diagnostic kits for testing patient samples. Initial testing was only available to Ochsner patients via the CDC RT-qPCR diagnostic assay performed by the Louisiana Office of Public Health (LA OPH). Because of the manual nature of the CDC assay and the sudden increase in volume, the LA OPH struggled with turnaround time and coordination of couriers.

Laboratory leadership at Ochsner—Dr Greg Sossaman, Dr Elise Occhipinti, Evelyn Smith, Earlene Goens, and Valinda Carmon—sought a better solution. Mayo Medical Laboratories was a logical option. Mayo Medical Laboratories has long provided quality results with clinically meaningful turnaround times for Ochsner, and with a robust courier process already in place, Mayo was well positioned to receive Ochsner samples. However, the nationwide demand quickly overwhelmed Mayo's capacity, forcing Mayo to send Ochsner specimens to other laboratories that were also struggling to meet demand. This divergence had a detrimental effect on turnaround time, with many patients waiting more than one week to receive their results.

Further, this delay led to a growing list of suspected COVID-19 patients, termed patients under investigation (PUIs), that critically strained Ochsner healthcare resources because PUIs required the use of enhanced personal protective equipment and negative pressure rooms—all of which were in scarce supply. Ochsner executive and laboratory leadership teams determined that insourcing SARS-CoV-2 testing was a necessity.

As laboratory leadership began strategizing how to insource testing, they quickly determined that a high throughput SARS-CoV-2 platform would be imperative because of the anticipated sample volume. Of the commercial assays pending FDA EUA approval, only two automated platforms met Ochsner criteria: Roche Diagnostics and Abbott Laboratories. By closely monitoring FDA EUA SARS-CoV-2 testing submissions, the molecular microbiology team of Dr Tong Yang, Dr Andrea Linscott, and Luke Caruso believed that Roche Diagnostics would be first to market with EUA approval. However, a few important points discouraged the team from going with the first available commercial test.

First, Ochsner had two Abbott instruments onsite, and staff was familiar with the analyzer functionality. Second, Ochsner had a long-standing partnership with Abbott Laboratories that was based on years of reliable chemistry assay performance, excellent service, and proven inventory distribution. Consequently, the team was extremely confident about Abbott's commitment to quality. Dr Tong Yang and Luke Caruso frequently communicated with Abbott scientific affairs staff; they knew the Abbott EUA approval was imminent and that the Abbott assay was developed using high-fidelity sequencing data. Last, Ochsner's Roche contacts disclosed that the Roche analyzers were on nationwide backorder, and reagents were allocated in such a way that Ochsner would be months out from receiving them. Consequently, the team decided to move forward with Abbott as Ochsner's primary commercial testing platform, even though Abbott received EUA approval nearly a week after Roche.

Ochsner obtained an advance shipment of Abbott testing kits for the SARS-CoV-2 RT-qPCR assay, allowing the molecular microbiology team to run internal verification tests on the Abbott instruments prior to EUA approval. Verification tests confirmed the accuracy, reproducibility, and limits of detection specific to SARS-CoV-2 to ensure confidence in our reporting. In addition, the team validated results using different transport mediums and swabs to account for the various sample collection kits—a safeguard that proved invaluable when shortages of specific swab types occurred. In parallel, the facilities management team put forth a Herculean effort to prepare for the arrival of the testing instruments and to install the necessary safety features. In less than 72 hours, the facilities team, led by Josh Bordelon and John Ferrara, made substantial upgrades to the designated SARS-CoV-2 testing room, creating a negative pressure testing environment, installing sufficient power outlets with emergency backups, and rerouting cable lines to ensure adequate information systems support. When complete, the space met the requirements for a Biosafety Level 2 laboratory.

Just 3 days after the FDA granted Abbott EUA approval and 11 days after our first COVID-19 patient admission, Ochsner went live on March 21, 2020 and became the first Louisiana hospital to run in-house diagnostic RT-qPCR testing for SARS-CoV-2.

But our work was not done. In mid-March, the number of cases in New Orleans was still increasing. We now faced the difficult task of scaling our testing capabilities to meet the growing pressures of the pandemic. We needed more staff and put out a call for volunteers to help in the lab.

The response was overwhelming. From chemistry, to blood bank, to microbiology, to pathology, scientists throughout Ochsner answered the call for help. Almost overnight, the SARS-CoV-2 laboratory transformed into a multidisciplinary team, united toward the common goal of curbing the pandemic. Simultaneously, other lab personnel took on extra work to ensure that the normal hospital workflow could continue uninterrupted. Karrie Hovis, education coordinator from the laboratory systemwide quality team, was instrumental in training redeployed clinical laboratory scientists. The demand for laboratory staff even went as high as the Governor of Louisiana who granted emergency clinical laboratory licenses for qualified medical students, which allowed University of Queensland-Ochsner Clinical School students Donald Chang and Guy Helman to volunteer. With the installation of a third Abbott instrument, testing capacity increased to more than 1,000 samples per day and, most importantly, patients’ test results could be available within one day.

This effort went beyond the walls of the laboratory. The Epic electronic medical record team, led by Matthew Doell, and laboratory information system teams, led by Cristina Guthrie, Wanda Eppling, and Stephanie Young, worked to quickly create test codes and test order sets and to make test reporting available.

As we worked to streamline our workflows and refine processes, we recognized the need to collaborate with and help other hospitals in the state establish in-house testing. Because of Ochsner's existing relationship with Louisiana State University (LSU) Health Shreveport, that institution was the natural choice for extending diagnostic testing capabilities to North Louisiana. An additional Abbott instrument was installed in the Ochsner LSU Shreveport molecular laboratory, allowing the site to begin testing patient samples on March 30, 2020—just 10 days after the Ochsner New Orleans laboratory went live.

We continued to expand our testing capacity by diversifying our testing platforms. While the Abbott high-throughput RT-qPCR assay could test more than 90 patient samples at once, it had the drawback of taking approximately 7 hours to run. On March 27, 2020, less than one week after going live with the Abbott high-throughput assay, the Ochsner microbiology team, led by Lauren Sensebe and April Davis, completed verification on the Cepheid GeneXpert testing platform that provided patient results in less than 1 hour.

One week later, on April 2, 2020, a collaborative effort between the microbiology and molecular laboratories resulted in the verification of the ID NOW testing platform from Abbott—a rapid SARS-CoV-2 assay with a 15-minute turnaround time. Drs Gregory Sossaman, Caroline Alquist, and Andrea Linscott joined Tammy Messer in traveling across the region to deliver materials and a plan for onsite staff to verify and implement the ID NOW platform at multiple hospitals throughout the system. The scope and rapidity of the ID NOW deployment was a significant milestone, as Ochsner became the first hospital system in the United States to offer this diagnostic test on such a large scale.

The addition of the Cepheid GeneXpert and ID NOW tests, coupled with the Abbott high-throughput assay, allowed us to increase our testing capacity substantially. The Ochsner SARS-CoV-2 laboratory could now confidently provide timely results for emergency department patients, all hospital admissions, and preoperative and preprocedural patients, as well as offer high-volume community screening.

Still, more needed to be done. Although the importance of diagnostic testing cannot be overstated, it became apparent that the epidemiologic and public health implications of COVID-19 could only be fully realized with antibody testing.

Led by Dr Qingli Wu and Debra Griener, the Ochsner chemistry laboratory worked around the clock to verify and implement the Abbott Laboratories SARS-CoV-2 immunoglobulin G (IgG) antibody testing platform. On April 22, 2020, one week after the antibody testing instruments were installed on April 15, 2020, Ochsner began offering serology testing to employees, and testing was extended to the community on May 4, 2020.

Supporting the frontline laboratory effort was the formation of several task forces, each with a unique focus centered around the testing workflow. Members of each task force were operational experts in their own areas and were managed by a dyad partnership between physician and administrator. For instance, a Laboratory Command Center led by Tammy Porter was created to field questions from community hospital laboratories and partner hospital laboratories. Daily dashboards showing specimen collection device and testing kit supply inventories were created and are still maintained by Warren Hovis of the Laboratory Command Center. Daily conference calls between physician leaders and laboratory directors and Evelyn Smith, vice president of the laboratory service line, escalated any issues related to logistics, supply procurement, or result interpretation. Independently, the task forces worked separately, but together, the efforts aligned to achieve success.

We offer three key takeaways from this experience. First, a hospital system must always be prepared for emerging healthcare challenges. While the pandemic did not significantly impact New Orleans until March, we started tracking the disease and developing a response months in advance. Second, the clinical laboratory and pathology are resource dependent. The success of the COVID-19 response is a good example of teamwork with strong administrative support. The laboratory team is grateful for how administration trusted our vision and provided the resources to make it possible. Last, cross-training staff, being open to a fluid schedule, and maintaining strict quality control are extremely important. Even with limited resources, tight time schedules, and high testing volumes, we did not lower our standards. Patient safety always comes first.

Our journey was not without hurdles. Despite new challenges every day, the Ochsner laboratory team rose to the occasion and met the demand of the communities we proudly serve. Ochsner staff—from system leadership to processing technicians—were eager to do whatever they could to provide timely and accurate diagnostics to our patients. Ochsner Chief Medical Officer Dr Robert Hart rolled up his sleeves and helped unpack boxes of supplies. Laboratory leaders, such as Director Earlene Goens and Manager Valinda Carmon, stepped out of the office and put on white lab coats and gloves to help define the workflow and prevent delays. The level of commitment from every staff member day in and day out was simply incredible.

None of this amazing work could have been done without the support of Ochsner administration leaders; members of the supply chain; information services staff; our vendor partners; laboratory physician and administrative leaders; PhD and MD supervisors for the molecular, microbiology, and chemistry laboratories; system clinical laboratory directors; and the dedicated medical laboratory professionals and medical students. In addition, without stable, robust, high-performing clinical laboratories already in place across the system, we could not have even attempted this endeavor.

